# MiR-1307-5p targeting TRAF3 upregulates the MAPK/NF-κB pathway and promotes lung adenocarcinoma proliferation

**DOI:** 10.1186/s12935-020-01595-z

**Published:** 2020-10-12

**Authors:** Xinyue Du, Shuangmiao Wang, Xingyan Liu, Tao He, Xiangui Lin, Simin Wu, Dan Wang, Jiao Li, Wenhua Huang, Huiling Yang

**Affiliations:** 1 School of Pharmacy, Guangdong Medical University, Zhanjiang, China; 2 National Key Discipline of Human Anatomy, School of Basic Medical Sciences, Southern Medical University, Guangzhou, China; 3 Affiliated Hospital of Guangdong Medical University, Zhanjiang, China; 4 Dongguan Scientific Research Center, Guangdong Medical University, Zhanjiang, China; 5 Department of Biology, School of Basic Medical Sciences of Guangdong Medical University, Guangzhou, China

**Keywords:** miRNA, Lung adenocarcinoma, TRAF3, NF-κb, MAPK

## Abstract

**Background:**

Non-small cell lung cancer (NSCLC) includes lung adenocarcinoma (LUAD) and lung squamous cell carcinoma (LUSC). MicroRNA (miRNA) plays an important role in the regulation of post-transcriptional gene expression in animals and plants, especially in lung adenocarcinoma.

**Methods:**

MiR-1307-5p is an miRNA with significant differences screened by the second generation of high-throughput sequencing in the early stage of our research group. In the current study, a series of in vitro and in vivo experiments were carried out. MiR-1307-5p mimic, miR-1307-5p inhibitor, and NC were transfected into A549 and H1299 lung adenocarcinoma cells. The correlation between miR-1307-5p and clinicopathological features in pathological samples was analyzed using a lung adenocarcinoma tissue microarray, and miR-1307-5p expression was detected by qPCR. CCK-8, EdU, colony formation, scratch test, and Transwell assays were used to observe cell proliferation and migration. Double luciferase assay, western blot, qPCR, and immunohistochemistry were employed in confirming the target relationship between miR-1307-5p and TRAF3. Western blotting was used to analyze the relationship between miR-1307-5p and the NF-κB/MAPK pathway. Finally, the effect of miR-1307-5p on tumor growth was studied using a subcutaneous tumorigenesis model in nude mice.

**Results:**

Increased miR-1307-5p expression was significantly related to decreased overall survival rate of lung adenocarcinoma patients, revealing miR-1307-5p as a potential oncogene in lung adenocarcinoma. MiR-1307-5p mimic significantly promoted while miR-1307-5p inhibitor reduced the growth and proliferation of A549 and H1299 cells. MiR-1307-5p overexpression significantly enhanced the migration ability while miR-1307-5p inhibition reduced the migration ability of A549 and H1299 cells. Target binding of miR-1307-5p to TRAF3 was confirmed by double luciferase assay, western blot, qPCR, and immunohistochemistry. miR-1307-5p caused degradation of TRAF3 mRNA and protein. MiR-1307-5p targeted TRAF3 and activated the NF-κB/MAPK pathway. TRAF3 colocalized with p65 and the localization of TRAF3 and p65 changed in each treatment group. Tumor volume of the lv-miR-1307-5p group was significantly larger than that of the lv-NC group, and that of the lv-miR-1307-5p-inhibitor group was significantly smaller than that of the lv-NC group.

**Conclusion:**

In conclusion, miR-1307-5p targets TRAF3 and activates the NF-κB/MAPK pathway to promote proliferation in lung adenocarcinoma.

## Background

Lung cancer is one of the leading causes of cancer-related deaths worldwide [1]. Pathologically, lung cancer is divided into two groups, non-small cell lung cancer (NSCLC) and small cell lung cancer (SCLC) [2]. NSCLC, including lung adenocarcinoma (LUAD) and lung squamous cell carcinoma (LUSC), accounts for approximately 85% of all lung cancers [3, 4]. Early research reported that the proportion of LUSC is higher than that of LUAD, and the proportion of LUSC among smokers is higher than that of LUAD [5, 6]. However, recent studies have reported that LUAD is the most common subtype in non-smoking lung cancer patients, and its incidence is significantly higher than that of LUSC [7]. Therefore, the significant increase in the proportion of LUAD in non-smoking lung cancer in recent years has led to a higher proportion of patients with LUAD than patients with LUSC, but the specific mechanism is still unclear [8]. Because the incidence of LUAD in non-smokers is increasing, the proportion of LUAD in lung cancer is the largest in recent years, so it is essential to discover the mechanisms underlying LUAD.

Chronic inflammation is a major risk factor for malignant transformation, tumor formation, tumor development, invasion, and metastasis in lung adenocarcinoma [9–11]. In recent years, it has been reported that NF-κB can participate in inflammatory and immune responses; regulate cell apoptosis, proliferation, and stress response; and act as a key promoter of tumor occurrence and other tumor responses [12, 13]. NF-kB regulates the expression of genes involved in many processes that play a key role in the development and progression of cancer such as proliferation, migration, and apoptosis. Aberrant or constitutive NF-kB activation has been detected in many human malignancies, and numerous studies have focused on elucidating the functional consequences of NF-kB activation as well as its signaling mechanisms [14]. NF-κB is continuously activated in many types of cancer, including colorectal, breast, lung, and some lymphomas [15–17]. Activation of the NF-κB pathway is related to the regulation of ovarian cancer mediated by TRIM52 (Tripartite Motif 52), which acts as a pro-cancer factor in ovarian cancer [18]. Prostate cancer patients usually carry the TMPRSS2/ERG (T/E) fusion gene, and T/E can activate the NF-κB pathway through phosphorylation of NF-κB p65 Ser536 (p536) to promote the occurrence and development of prostate cancer [19]. NF-κB is upregulated in lung adenocarcinoma and precancerous lesions, and its activation is associated with poor prognosis in lung adenocarcinoma patients [20]. Finally, activated NF-κB (especially p65) is induced by p53 deletion or the expression of SQSTM1 (Sequestosome 1) induced by the oncogene KRAS, which plays an important role in KRAS (G12D)-induced lung tumor growth [21].

MiRNAs are small non-coding regulatory RNAs that bind to the 3′ untranslated region (3′ UTR) of mRNA and regulate its function and that of endogenous genes by influencing the translation process [22, 23]. Dysregulation of miRNA expression is associated with abnormal gene expression and is involved in the development of lung adenocarcinoma, such as cell proliferation, apoptosis, migration, invasion, and differentiation [24, 25]. In this study, we selected miR-1307-5p from among 40 different miRNA expression profiles that we had screened by second-generation high-throughput sequencing after p65 transfection of A549 cells to investigate the mechanism of miR-1307-5p regulation in lung adenocarcinoma [26]. MiR-1307-5p and miR-1307-3p were obtained by modifying the 5′ telomere and 3′ telomere of the precursors of miR-1307. Previous studies have shown that miR-1307 may promote the proliferation of prostate cancer cells by targeting FOXO3A [27], that downregulation of SMYD4 by miR-1307-3p can influence the occurrence and development of breast cancer cells [28], and that miR-1307-3p affected the growth and metastasis of hepatocellular carcinoma by inhibiting the DAB2 interacting protein [29]. However, the role of miR-1307-5p in lung adenocarcinoma and its molecular mechanism remains unclear.

In this study, we found that miR-1307-5p may play a regulatory role with TRAF3 (TNF Receptor Associated Factor 3), which is a protein-coding gene of the TNF receptor associated factor (TRAF) protein family [30]. Among its related pathways are the metabolism of proteins and the Toll-like Receptor signaling pathway [31]. TRAF3 regulates pathways leading to the activation of NF-κB and MAP kinases and plays a central role in the regulation of B-cell survival [32]. TRAF proteins associate with, and mediate signal transduction from, TNF receptor (TNFR) superfamily members [33]. TRAF3 participates in the signal transduction of CD40, a TNFR family member important for the activation of the immune response [34]. CD40 is a critical component of the lymphotoxin-beta receptor (LTbetaR) signaling complex, which induces NF-κB activation and cell death initiated by LTbeta ligation [35, 36]. This study will explore in detail the regulatory role of miR-1307-5p in inflammation and lung adenocarcinoma.

## Methods

### Clinical specimens

Tissue samples were obtained from 15 patients with LUAD treated surgically in the oncology department of Integrated Traditional Chinese and Western Medicine Hospital, Southern Medical University (Guangzhou, China). All human materials were obtained with informed consent, and this study was approved by the research ethics committee of the Integrated Traditional Chinese and Western Medicine Hospital of Southern Medical University in Guangzhou.

A LUAD tissue chip was purchased from Shanghai Xinchao Biotechnology Co., Ltd. (Shanghai, China). The tissue chip contained 180 cores, with 90 paraffin-embedded primary LUAD specimens and 90 adjacent normal lung specimens from patients. Clinical and statistical data were obtained from the patients’ medical records.

### Presentation data set

The putative relationship between miR-1307-5p and TRAF3 was predicted using TargetScan (https://www.targetscan.org/).

### Experimental animals

BALB/c-nu nude mice were purchased from Jiangsu Jihui Yaokang Biotechnology Co., Ltd.(Jiangsu, China), animal qualification certificate number 201904423. The experiments involving animals were approved by the Ethics Committee for the Use and Care of Animals of Southern Medical University(Guangzhou, China).

### Cell lines

Human A549 and H1299 lung adenocarcinoma cells used in the study were primarily obtained from American Type Culture Collection (ATCC, Manassas, VA, USA). Human normal lung 16HBE cells were obtained from NTCC Preservation Center (Beijing, China).

### Cell culture and transfection

Human A549 and H1299 cells were cultured in 1640 medium containing 10% FBS (Gibco, USA), human normal lung 16HBE cells were cultured in KM medium (Sciencell, USA), and incubated at 37 °C in a 5% CO_2_ incubator (Nikon, Japan). Cells with good logarithmic phase growth and no contamination were selected for the experiment. Transient transfection: miR-1307-5p ((5′- > 3′): UCGACCGGACCGGCU; (3′- > 5′): AGCUGGCCUGGAGCUGGCCGA), miR-1307-5p inhibitor ((5′- > 3′): AGCCGGUCGAGGUCCGGUCGA) and miR-NC (Guangzhou Reibo Biotechnology Co., Ltd., Guangzhou, China) were transfected with Lipofectamine® RNAiMAX Reagent (Invitrogen, USA) at appropriate time points. Lentiviral transfection: After inoculation in a 24-well plate at a density of 8 × 10^3^/well and 96 h for transfection following the instructions for the lentiviral reagent (Shanghai JiKaiJi for Science and Technology Co., Ltd., Shanghai, China), the cells remained in culture containing the appropriate concentration of puromycin (Cayman, USA) until all non-transfected cells were killed but none of the transfected cells. Finally, the hybrid clonal stable strains were screened.

### Real-time quantitative PCR

Cells were grown in six-well plates (Corning, USA) from an initial cell density of 2 × 10^5^ cells/well. At 48 h after transfection, the Trizol method (Invitrogen, USA) was used to obtain total RNA, then cDNA synthesis was performed by reverse transcription PCR (Invitrogen, USA) using the SYBRGreen method (Thermo Fisher Scientific, USA) and a PCR-7500 real-time PCR system (Applied Biosystems, USA). The internal parameters were uniformly normalized by RNU6B(U6) and the relative quantitative calculation of multiple changes used the 2^−ΔΔCq^ method. The following primers (Shanghai Bioengineering Co., LTD., Shanghai, China) were used: hsa-miR-1307-5p, forward: CGGGCTCGACCGGACCTCG, reverse: CAGCCACAAAAGAGCACAAT; stem loop sequence (used for reverse transcription): CCTGTTGTCTCCAGCCACAAAAGAGCACAATATTTCAGGAGACAACAGGAGCCGGT; U6, forward: CTCGCTTCGGCAGCACA, reverse: AACGCTTCACGAATTTGCGT; stem loop sequence (for reverse transcription): AACGCTTCACGAATTTGCGT; TRAF3, forward: TCTTGAGGAAAGACCTGCGAG, reverse: GCGATCATCGGAACCTGACT; GAPDH, forward: CGCTGAGTACGTCGTGGAGTC, reverse: GCTGATGATCTTGAGGCTGTTGTC.

### Western blot (WB) analysis

After 72 h incubation with an initial cell density of 2 × 10^5^ cells/well, total protein was extracted. After SDS-PAGE electrophoresis, membrane transfer, and blocking, the membrane was incubated overnight with primary antibodies (TRAF3 (cat. no. 66310-1-Ig, ProteinTech Group, USA), p65 (cat. no. #8242S, Cell Signaling Technology, USA), p-p65 (cat. no. #3033S, Cell Signaling Technology, USA), ERK1/2 (cat. no. 11257-1-AP, ProteinTech Group, USA), p-ERK1/2 (cat. no. #4370S, Cell Signaling Technology, USA), p38 (cat. no. #8690S, Cell Signaling Technology, USA), p-p38 (cat. no. #4511 T, Cell Signaling Technology, USA), tubulin (cat. no. #5335S, Cell Signaling Technology, USA), actin (cat. no. 66009-1-Ig, ProteinTech Group, USA)) and then with goat-anti-mouse/rabbit secondary antibody (cat. no. #7076S, #7074S, Cell Signaling Technology, USA). Finally, the signal of the immobilized target proteins on the membranes was developed by enhanced chemiluminescence (ECL) using the ECL Reagent (Merck Millipore, Darmstadt, Germany). Images were captured with a Chemiluminescence Imager (Sage Creation Science Co., Ltd., Beijing, China).

### Proliferation assay

The cells were inoculated into 96-well plates at a density of 3 × 10^3^ cells/well. After transfection, the cells were incubated for another 24 h. Twenty μl of CCK-8 reagent (Dojindo Company, Japan) was added to each well at three time points (24, 48, 72 h). Cells were gently shaken and incubated for 2 h. A microplate reader (BioTek, USA) was then used to measure the optical density of each well at the wavelength of 450 nm and the cell growth curve was then drawn.

### Colony formation assay

To 200 cells/well in six wells, we added 2 ml of 1640 culture medium containing 10% FBS and cultured at 37 °C in 5% CO_2_ for about 2 wk to permit cell colony formation. After 3 PBS washes, the cells were fixed in 1 ml of 4% paraformaldehyde (Beijing Solabao Company, Beijing, China) for 15 min. The fixative was then discarded, and the cells were washed three times with PBS. Staining used 1 ml/well of crystal violet solution (Jiangsu Biyuntian Company, Jiangsu, China) for 5 min and photos were taken. The number of clone colonies in each group was counted and analyzed statistically.

### EdU proliferation assay

The transfected cell suspensions were seeded in 96-well plates with a density of 8 × 10^3^ cells/well and cultured in a cell incubator with 37 °C and 5% CO_2_ for 24 h. EdU labeling, cell immobilization, Apollo staining, and DNA staining were carried out according to the manufacturer’s EdU Kit instructions (Guangzhou Ruibo Biotechnology Co., Ltd., Guangzhou, China), and cell fluorescence intensity was observed by fluorescence microscope (Japan Olympus Co., Ltd., Tokyo, Japan) and subjected to photo analysis.

### Transwell migration assay

We added 1640 medium containing 10% FBS (600 μL) to the lower compartment of each well in a 24-well plate(Corning, USA), then placed a Transwell insert in each well. Then 200 μL of 8 × 10^3^ cells/well were added into the cell suspension, after which the plates were incubated for 24 h. The chambers were then removed, wiped gently with a cotton swab to remove cells that did not pass through the membrane, washed 3 times in PBS, and then fixed with 4% paraformaldehyde for 15 min. After removing the fixative and rinsing 3 times with PBS, staining with crystal violet solution was carried out for 5 min. At least 5 sites in each chamber were selected for photomicrography and the number of cells that passed through the membrane was calculated for statistical analysis.

### Scratch migration assay

After transfection, cells were cultured in 1640 culture medium containing 10% FBS in the six-well plate with a density of 3 × 10^5^ cells/well until the cells were 90% confluent. A straight mark was scratched across the middle of each pore with a 100 μl pipette tip. Upper, middle, and lower locations along each scratch were photographed at 0, 24, 48, and 72 h, and scratch widths were recorded to compare the healing rate for each group.

### Double luciferase assay

The wild-type (WT) and mutant (MUT) miR-1307-5p binding sites in the 3′ UTR of TRAF3 were cloned into the psicheck promote reporter gene vector (Promega Company, USA) to construct the luciferase plasmid. The experimental groups were blank control group, no-load control group, wild-type group, and mutation group; miRNA groups were NC, mimic, and inhibitor (Guangzhou Ruibo Biotechnology Co., Ltd., Guangzhou, China). For the reporter gene assay, the psicheck promote reporter gene vector and miR-1307-5p analog were co-transfected with EndoFectin Lenti reagent. Following the manufacturer’s instructions, luciferase activity was measured 24 h later with a dual luciferase reporter kit (with firefly luciferase as the reporter gene and *Renilla* luciferase as the internal reference gene), and the data were standardized for *Renilla* luciferase activity.

### In vivo xenograft model

Pathogen-free BALB/c-nu thymus-free nude mice (4–5 weeks old, male) were used, with two groups of five mice each (groups lv-miR-1307-5p and lv-miR-1307-5p inhibitor, each compared with lv-NC in the same animal). The overexpression stable strain and negative control stable strain cells were separately inoculated subcutaneously to the left and right sides of one group of mice (5 × 10^6^ = number of cells injected per nude mouse), and the inhibition stable strain and negative control stable strain cells were separately inoculated subcutaneously to the left and right sides of the other group of mice (5 × 10^6^ = number of cells injected per nude mouse). Eight days later, tumors were established. Tumor size and body mass were measured every five days. The volume was calculated as (length × width × width)/2, and the tumor tissue was collected for analysis.

### Cellular immunofluorescence

Cells were inoculated at a density of 4 × 10^4^ and incubated in a laser confocal culture dish (Corning, USA) for 24 h. After fixation with 4% paraformaldehyde and treatment with 0.5% Triton X-100, the dish was closed at room temperature and incubated with Alexa Fluor®conjugated secondary antibody (goat anti-mouse IgG H&L Alexa fluor®647, ab150115; goat anti-rabbit IgG H&L Alexa fluor®488, ab150077;abcam, USA) at 4 °C overnight, incubated with fluorescent second antibody for 1 h, stained with DAPI, sealed with the sealing solution containing anti-fluorescent quenching agent, and then observed under the fluorescence microscope.

### Immunohistochemistry

Tumor tissue was fixed with 4% paraformaldehyde, embedded in paraffin, sectioned, and used for immunohistochemistry. The sections underwent antigen retrieval in CPBS buffer, and goat serum was added dropwise to the tissue for blocking at room temperature, then primary antibody to TRAF3 (1: 200) was added dropwise and incubated overnight at 4 °C. The next day, DAB color developing solution (Dalian Meilun Biotechnology Co., Ltd., Dalian, China) was used for color development, followed by counterstaining with hematoxylin (Wuxi Jiangyuan Industrial Technology & Trade Corporation, Wuxi, China). The slides were then dehydrated, dried at room temperature, and sealed with neutral resin.

### miRNA in situ hybridization

Lung adenocarcinoma tissue chip was dewaxed, rehydrated through a descending ethanol gradient, then treated with 0.1% hydrochloric acid–ethanol for 15 min. The tissue chip was incubated with diluted pepsin in 3% fresh citrate buffer at 37 °C for 20 min and then washed with phosphate-buffered saline (PBS). The chip was fully digested to expose the mRNA of the tissue on the chip, which can enhance the subsequent immune hybridization signal. The digoxin-labeled miRNA probe (Biosense Bioscience Co. Ltd., Guangzhou, China) was diluted with the hybridization diluent and hybridized overnight at 37 °C. Blocking solution was added dropwise, DAB staining solution was used for color development, and hematoxylin counterstaining was performed. Slides were dehydrated, dried at room temperature, and sealed with neutral resin.

### Statistical analysis

The data were analyzed using SPSS 20.0 (SPSS, USA) or GraphPad Prism 6 (GraphPad Prism, USA). Every experiment was completed independently at least three times. A *P* value < 0.05 was considered significant.

## Results

### MiR-1307-5p is upregulated in LUAD

**Fig. 1:**
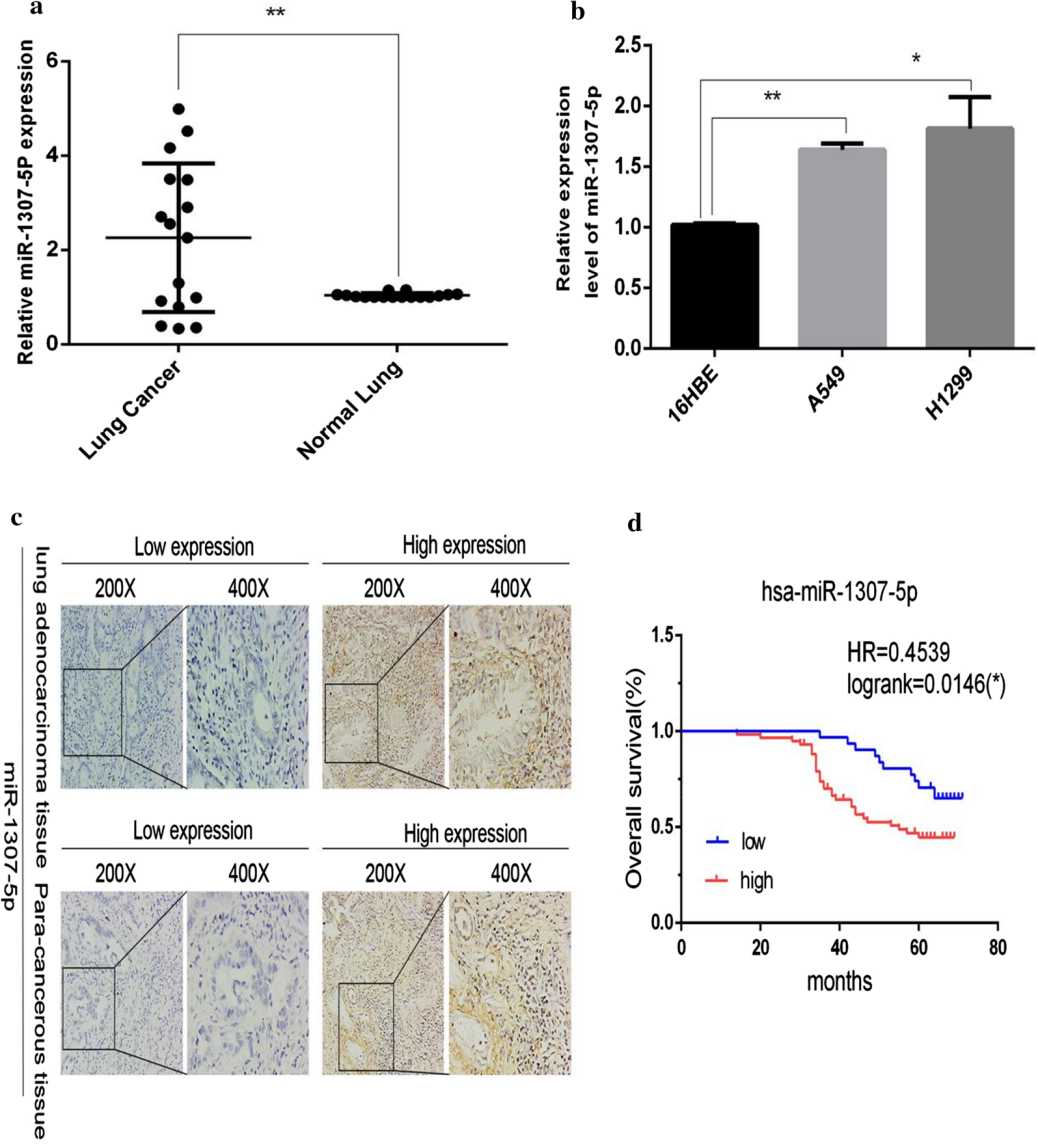
MiR-1307-5p was upregulated in LUAD. **a** QPCR showed the relative expression level of miR-1307-5p in lung adenocarcinoma and adjacent tissues. Compared with the control group, *P < 0.05 and **P < 0.01. **b** QPCR showed the relative expression of miR-1307-5p in normal human lung cells (16HBE) and two lung adenocarcinoma cell lines (A549, H1299). Compared with 16HBE cell line, *P < 0.05 and **P < 0.01. **c** The expression of miR-1307-5p in lung adenocarcinoma and nearby paracancerous tissue was detected by in situ hybridization. **d** The increase of miR-1307-5p is in direct proportion to the decrease in overall survival rate of lung adenocarcinoma. Data are expressed as mean ± standard deviation. The experiment was repeated three times

The expression level of miR-1307-5p detected by real-time fluorescence quantitative PCR indicated that miR-1307-5p was significantly upregulated in most lung adenocarcinoma tissues compared with neighboring paracancerous tissue (Fig. 1a). Subsequently, we evaluated miR-1307-5p in cultured normal human lung epithelial cells (16HBE) and two lung adenocarcinoma cell lines (A549, H1299). MiR-1307-5p expression levels in the lung adenocarcinoma cells were significantly upregulated compared to normal 16HBE (Fig. 1b). In situ hybridization showed that expression of miR-1307-5p was upregulated in lung adenocarcinoma, and the increase of miR-1307-5p was significantly related to the decrease of overall survival rate in lung adenocarcinoma. In the pathological analysis of lung adenocarcinoma, patients with low miR-1307-5p expression had a more favorable prognosis (*P* = 0.0146) (Fig. 1c, d; Table 1). MiR-1307-5p was closely related to tumor size (T stage) (*P* = 0.027), but not to age, gender, pathological type, pathological stage, lymph node metastasis (N stage), distant metastasis (M stage), or clinical stage (Table 2).

**Table 1:** detection of mir-1307-5p expression in cancer and adjacent tissues by in situ hybridization

Group	n	Expression level of mir-1307-5p		
		Low expression	High expression	*P*
Adenocarcinoma of lung	90	31 (34.4%)	59 (65.6%)	
				0.002
Paracancerous tissue	90	52 (57.8%)	38 (42.2%)

**Table 2:** relationship between expression of mir-1307-5p and clinicopathological factors

Pathological parameters	n	Expression level of mir-1307-5p		
		high expression	Low expression	*P*
Age				
<55	28	18 (30.5%)	10 (32.3%)	
				0.524
≥ 55	62	41 (69.5%)	21 (67.7%)	
Gender				
Male	46	34 (57.6%)	12 (38.7%)	
				0.069
Female	44	25 (42.4%)	19 (61.3%)	
Pathological stage				
I	15	10 (16.9%)	5 (16.1%)	
II	55	36 (61.0%)	19 (61.3%)	0.994
III	20	13 (22.0%)	7 (22.6%)	
TNM staging				
T				
T1	65	47 (79.7%)	18 (58.1%)	
T2	21	11 (18.6%)	10 (32.3%)	
				0.027
T3	3	0 (0.0%)	3 (9.7%)	
T4	1	1 (1.7%)	0 (0.0%)	
N				
N0	56	36 (61.0%)	20 (64.5%)	
N1	21	15 (25.4%)	6 (19.4%)	
				0.797
N2	13	8 (13.6%)	5 (16.1%)	
N3	0	0 (0.0%)	0 (0.0%)	
M				
M0	90	43 (72.9%)	22 (71.0%)	
				0.517
M1	2	16 (27.1%)	9 (29.0%)	
Clinical stages				
I	40	25 (42.4%)	15 (48.4%)	
II	19	15 (25.4%)	4 (12.9%)	
				0.504
III	6	3 (5.1%)	3 (9.7%)	
IV	25	16 (27.1%)	9 (29.0%)	

### MiR-1307-5p promotes the proliferation of LUAD cells

**Fig. 2:**
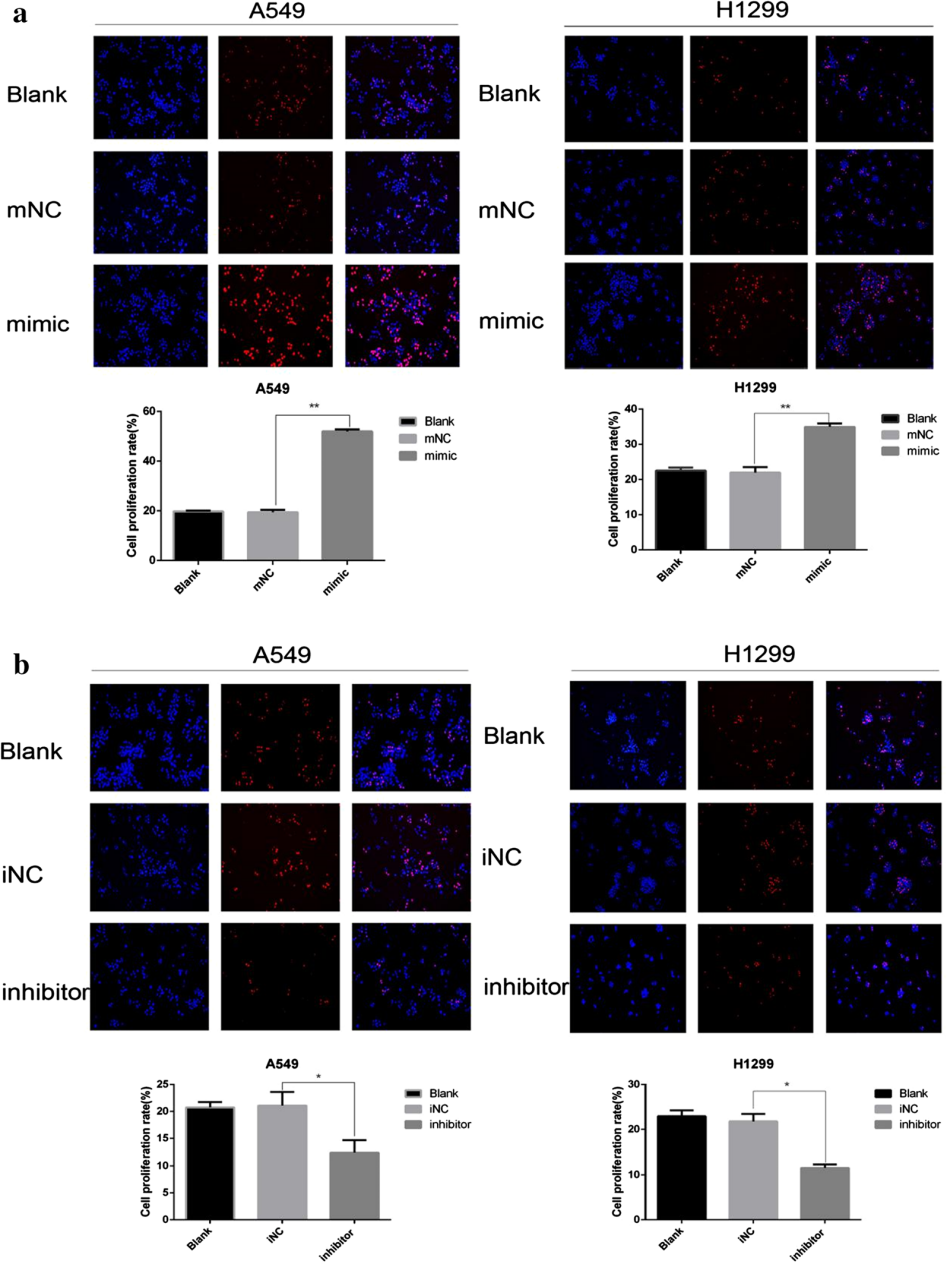
MiR-1307-5p promotes the proliferation of LUAD cells. **a**, **b** EdU was used to detect the proliferation of H1299 and A549 lung adenocarcinoma cells after transient transfection. Overexpression of miR-1307-5p promoted cell proliferation (**a**), while knockdown of miR-1307-5p inhibited cell proliferation (**b**). Compared with the control group, *P < 0.05 and **P < 0.01. Data are expressed as mean ± standard deviation. The experiment was repeated three times

A549 and H1299 cells were transfected with miR-NC, miR-1307-5p mimic, or miR-1307-5p inhibitor. The expression of miR-1307-5p was examined by qPCR at 48 h after transfection. Compared with miR-1307-5p mimic transfection, miR-1307-5p expression was significantly increased (Additional file 1: Figure S1A), while miR-1307-5p inhibitor transfection reduced the expression of miR-1307-5p (Additional file 1: Figure S1B). CCK-8 analysis of cell proliferation was used to further investigate the effect of miR-1307-5p overexpression or downregulation on the proliferation of lung adenocarcinoma cells. The proliferation of lung adenocarcinoma cells overexpressing miR-1307-5p was significantly higher than that of the control group (Additional file 1: Figure S1C). Conversely, knockdown of miR-1307-5p resulted in reduced proliferation of lung adenocarcinoma cells compared to the control group (Additional file 1: Figure S1D). By EdU cell proliferation assay, the proliferation rate of lung adenocarcinoma cells transfected with miR-1307-5p mimic was significantly higher than that of the control group (Fig. 2a) and the proliferation rate of lung adenocarcinoma cells transfected with miR-1307-5p inhibitor was lower than that of the control group (Fig. 2b). These results suggest that miR-1307-5p plays an important role in regulating the proliferation of lung adenocarcinoma cells.

### MiR-1307-5p promotes the migration of LUAD cells

**Fig. 3:**
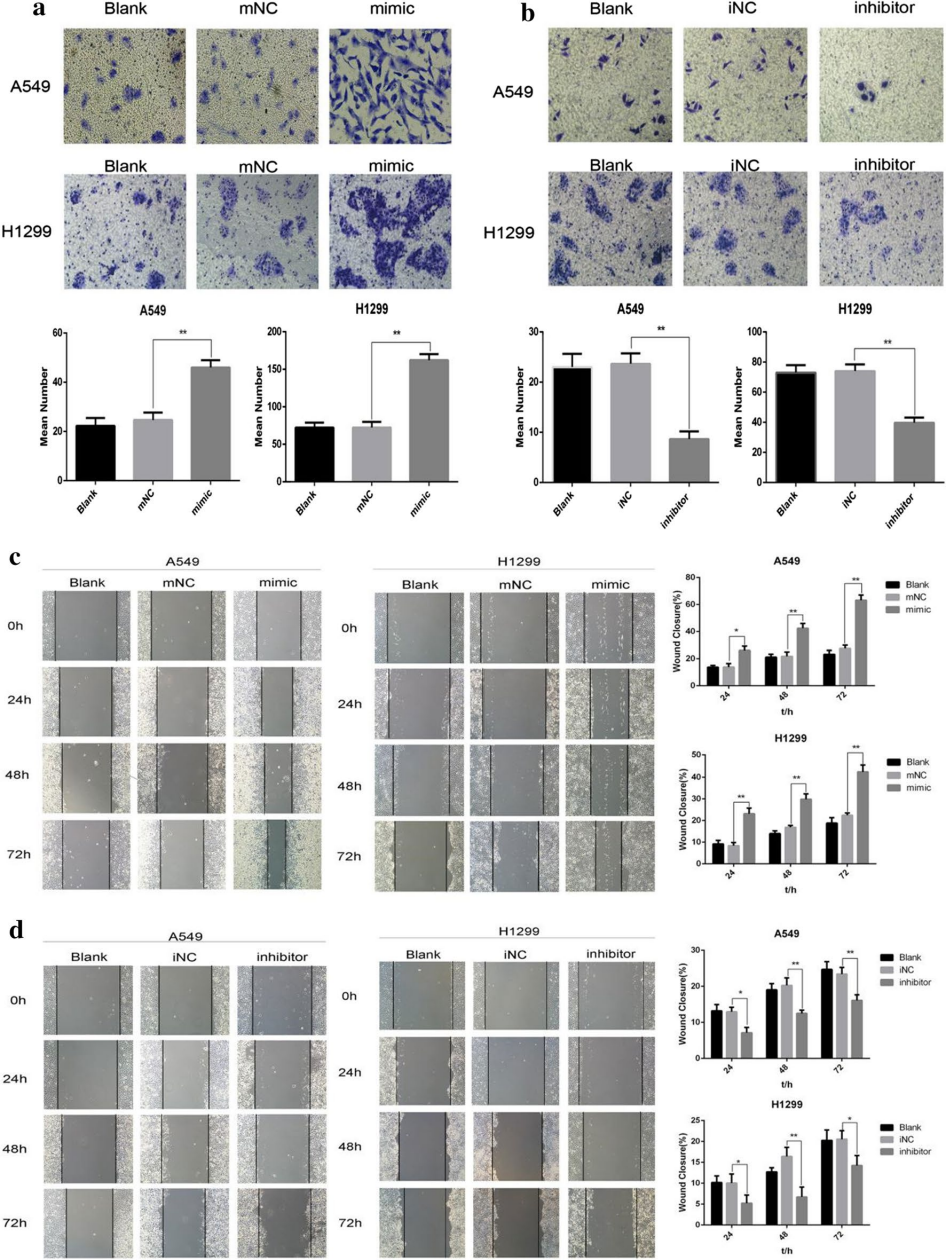
MiR-1307-5p promotes the migration of LUAD cells. **a**, **b** The Transwell test was used to detect the migration of each treatment group after transient transfection of H1299 and A549 lung adenocarcinoma cells. Overexpression of miR-1307-5p promoted cell migration (**a**), while knockdown of miR-1307-5p inhibited cell migration (**b**). Compared with the control group, * *P* < 0.05 and ** *P* < 0.01. **c**, **d** The scratch test was used to detect the migration and healing of each treatment group after transient transfection of H1299 and A549 lung adenocarcinoma cells. Overexpression of miR-1307-5p promoted cell migration (**c**), while knockdown of miR-1307-5p inhibited cell migration (**d**). Compared with the control group, *P < 0.05 and **P < 0.01. Data are expressed as mean ± standard deviation. The experiment was repeated three times

A549 and H1299 cells were transfected with miR-NC, miR-1307-5p mimic, or miR-1307-5p inhibitor. In the Transwell assay, miR-1307-5p promoted the migration of lung adenocarcinoma cells in the mimic group compared to the control group (Fig. 3a), while downregulation of miR-1307-5p significantly inhibited migration (Fig. 3b). These results were consistent with the scratch experiment, in which miR-1307-5p promoted migration in the mimic group compared with the control group (Fig. 3c), whereas downregulation of miR-1307-5p significantly inhibited migration (Fig. 3d).

### TRAF3 is a target of miR-1307-5p in LUAD cells

**Fig. 4:**
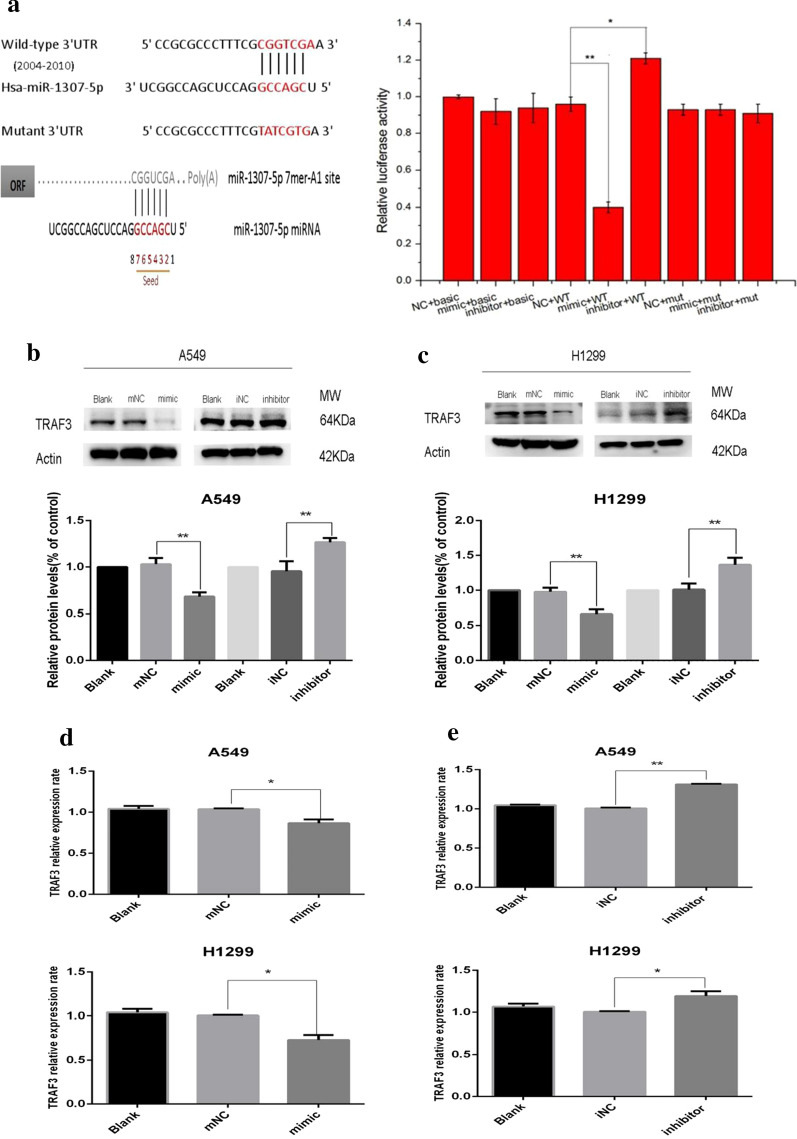
TRAF3 is a direct target of miR-1307-5p in LUAD cells. **a** The binding degree of miR-1307-5p to TRAF3 was detected by double luciferase assay. MiR-1307-5p targets TRAF3. Compared with the control group, *P < 0.05 and **P < 0.01. **b**, **c** Western blot was used to detect the difference of TRAF3 protein levels in the treatment groups after transient transfection of H1299 and A549 lung adenocarcinoma cells. Overexpression of miR-1307-5p downregulated the protein expression level of TRAF3 (**b**) and inhibited the upregulated protein expression level of TRAF3 (**c**). Compared with the control group, *P < 0.05 and **P < 0.01. **d**, **e** QPCR was used to detect the difference of TRAF3 mRNA level between the treatment groups after transient transfection of H1299 and A549 lung adenocarcinoma cells. Overexpression of miR-1307-5p downregulated the mRNA expression level of TRAF3 (**d**) and inhibited the upregulated mRNA expression level of TRAF3 (**e**) by miR-1307-5p. Compared with the control group, *P < 0.05, **P < 0.01 and ***P < 0.005. Data are expressed as mean ± standard deviation. The experiment was repeated three times

Through bioinformatics analysis, the potential targets of miR-1307-5p in lung adenocarcinoma were further investigated. It has been predicted that TRAF3 is the direct target of miR-1307-5p and that the target relationship is evolutionarily conserved. To confirm this, wild-type or mutant subclones of miR-1307-5p binding sequences from the TRAF3 3′ UTR were cloned into pmirGLO vectors downstream of the firefly luciferase reporter gene, named v80-pmirglo-traf3 WT and v80-pmirglo-traf3 mut, and co-transfected into 293 T cells with miR-1307-5p mimic, miR-1307-5p inhibitor, or miR-NC. Luciferase reporter gene assay was performed 24 h after transfection. The results showed that NC, mimic, and inhibitor had no effect on the mutant vector and no-load activity of the target gene. The mimic significantly inhibited the activity of wild-type vector of the target gene, whereas the activity of wild-type vector of the target gene was improved after Inhibitor treatment. After site mutation, the mimic had no inhibitory effect on the mutant target gene vector, and after the inhibitor treatment, and the activity of the mutant vector was also not affected (Fig. 4a). By western blot analysis, TRAF3 protein expression was downregulated in the mimic group compared with the control group (Fig. 4b, c) and upregulated in the inhibitor group compared with the control group (Fig. 4b, c). Meanwhile, qPCR analysis showed that TRAF3 mRNA expression was downregulated in the mimic group compared with the control group (Fig. 4d, e) and upregulated in the inhibitor group compared with the control group (Fig. 4d, e). The results showed that miR-1307-5p could bind directly to the 3′ UTR of TRAF3 mRNA.

### MiR-1307-5p targets TRAF3 to regulate the NF-κB/MAPK pathway

**Fig. 5:**
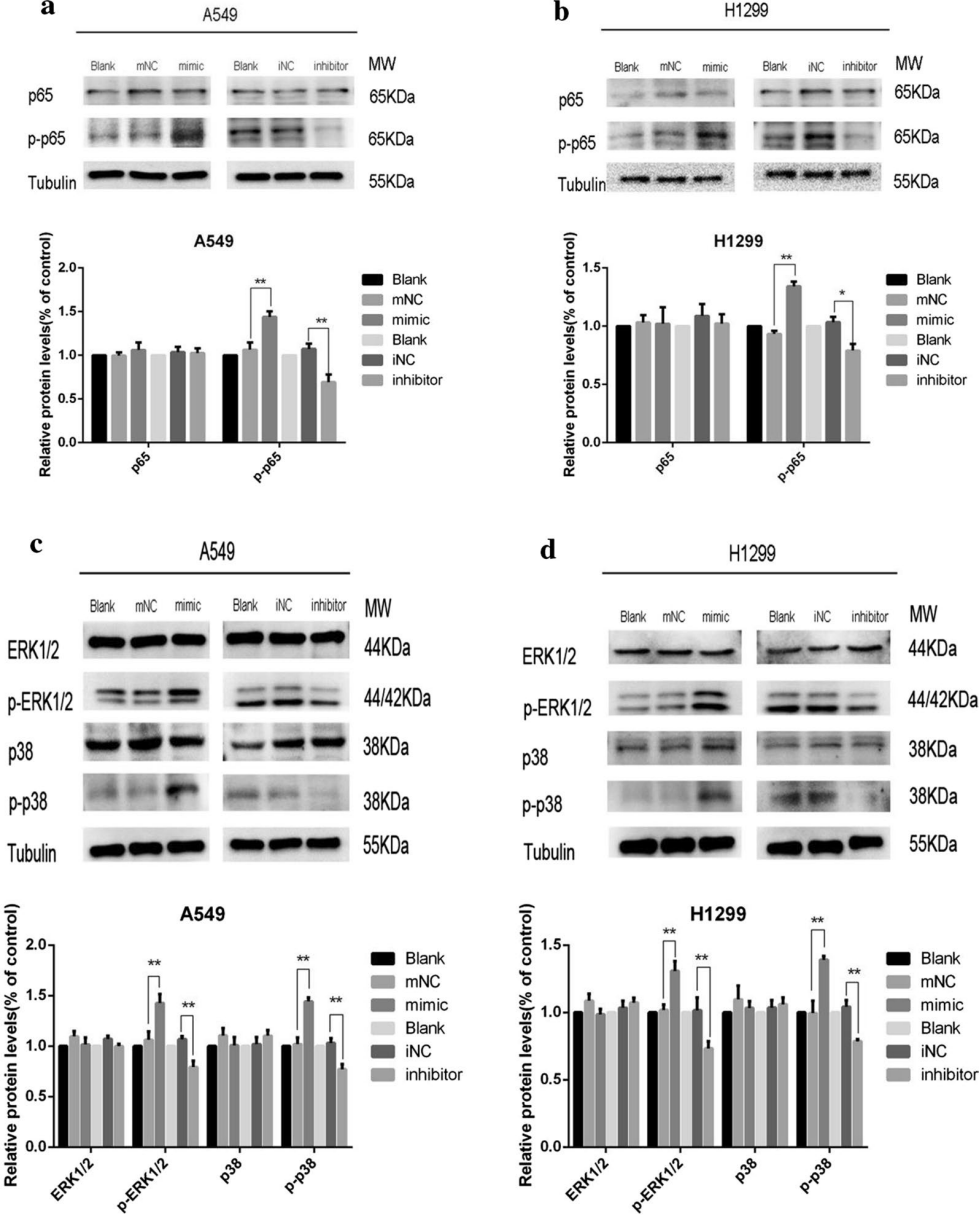
MiR-1307-5p targets TRAF3 to regulate the NF-κB/MAPK pathway. **a**, **b** Western blot was used to detect the difference of NF-κB pathway protein levels in each treatment group after transient transfection of H1299 and A549 lung adenocarcinoma cells. Overexpression of miR-1307-5p upregulated p-p65 protein expression level and inhibited miR-1307-5p downregulated p-p65 protein expression level. Compared with the control group, *P < 0.05 and **P < 0.01. **c**, **d** Western blot was used to detect the difference of MAPK pathway protein levels in the treatment groups after transient transfection of H1299 and A549 lung adenocarcinoma cells. Overexpression of miR-1307-5p activated the MAPK pathway and knockdown of miR-1307-5p inhibited the MAPK pathway. Compared with the control group, *P < 0.05 and **P < 0.01. Data are expressed as mean ± standard deviation. The experiment was repeated three times

After transfection with NC, mimic, and inhibitor, western blot showed that the p-p65 protein level in the mimic group was significantly upregulated compared with the control group (Fig. 5a, b), whereas it was significantly decreased in the inhibitor group (Fig. 5a, b). In the cell immunofluorescence study, the co-localization of p65 and TRAF3 was significant. Compared with the control group and the blank group, lung adenocarcinoma cells in the mimic group showed lower expression of cytoplasmic TRAF3 while p65 fluorescence was enhanced and expressed in both the cytoplasm and the nucleus (Additional file 2: Figure S2A). Conversely, TRAF3 fluorescence was relatively increased in the inhibitor group and expressed in the cytoplasm and partially in the nuclei, while p65 fluorescence was decreased and only partially expressed in the cytoplasm (Additional file 2: Figure S2B). The results showed that miR-1307-5p targeting TRAF3 activates the NF-κB pathway. Compared with the control group, the protein levels of p-ERK1/2 and p-p38 in the mimic group were significantly upregulated (Fig. 5c, d), whereas the protein levels of P-ERK1/2 and P-P38 in the inhibitor group were significantly decreased relative to controls (Fig. 5c, d). These results indicate that miR-1307-5p targeting TRAF3 activates the MAPK pathway.

### MiR-1307-5p promoted the proliferation of stable lentivirus strains of LUAD

The overexpression, inhibition, and negative control lentivirus stable strains were constructed and the expression of miR-1307-5p was detected by qPCR. The expression of miR-1307-5p was significantly increased in the miR-1307-5p overexpression group (Additional file 3: Figure S3A) and decreased in the miR-1307-5p inhibition group (Additional file 3: Figure S3A). In the clone formation experiment, the lung adenocarcinoma cell clone proliferation rate of the miR-1307-5p overexpression group was significantly higher than that of the control group (Additional file 3: Figure S3B), whereas the rate of the miR-1307-5p inhibition group was lower than that of the control group (Additional file 3: Figure S3B). The effects of miR-1307-5p overexpression or downregulation on the proliferation of lung adenocarcinoma cells were further studied by CCK-8 analysis. Here, the proliferation of miR-1307-5p overexpressed stable lung adenocarcinoma strains was significantly higher than that of the control group (Additional file 3: Figure S3C), while miR-1307-5p inhibited the proliferation of lung adenocarcinoma cell lines in comparison with the control group (Additional file 3: Figure S3C). These results indicate that miR-1307-5p can promote the proliferation of lung adenocarcinoma cells.

### MiR-1307-5p promotes tumor growth in vivo

**Fig. 6:**
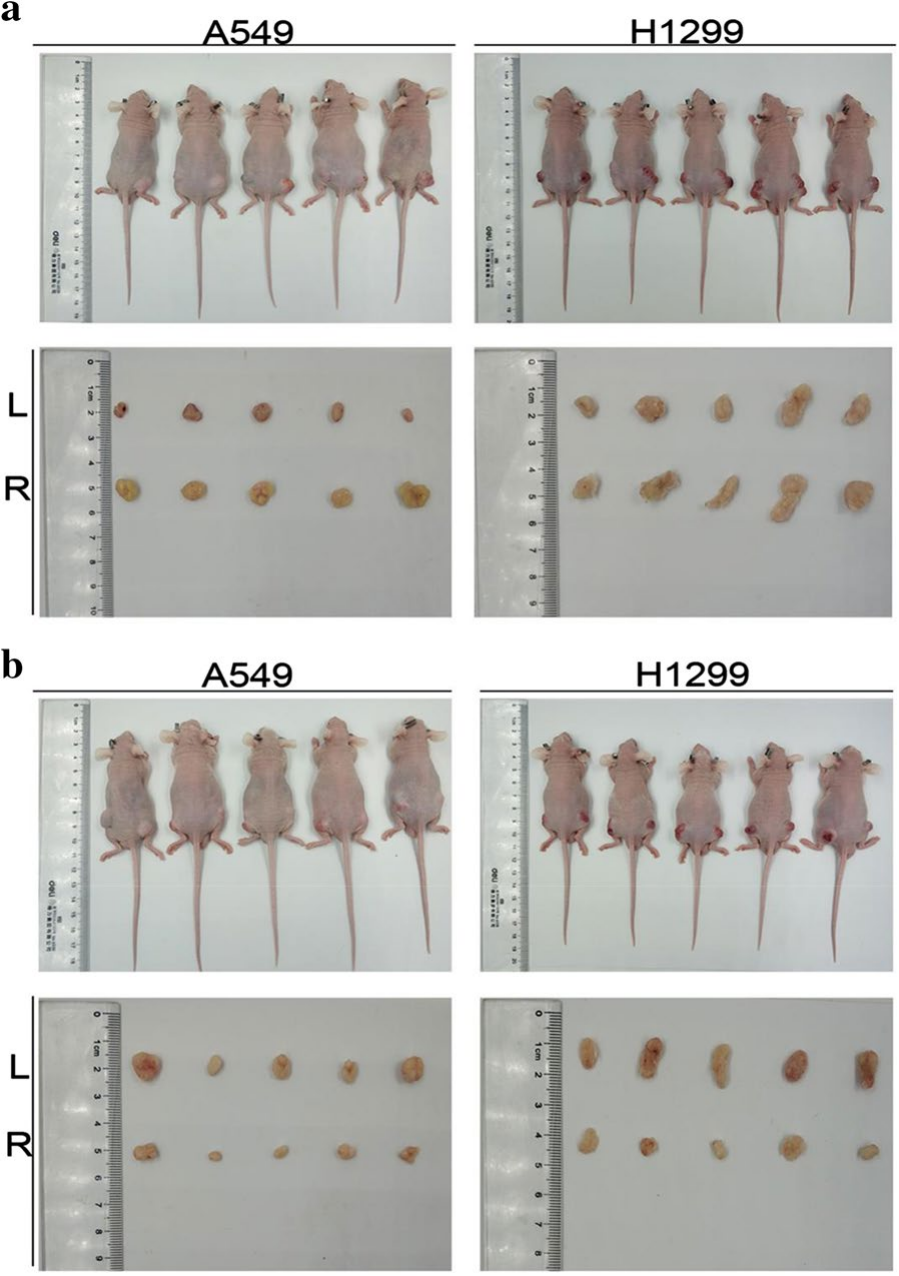
MiR-1307-5p promotes tumor growth in vivo. **a** A549 and H1299 cells were inoculated subcutaneously in nude mice after stable overexpression of miR-1307-5p. On the 20th day; L (left) is lv-NC, R (right) is lv-miR-1307-5p. **b** A549 and H1299 cells were inoculated subcutaneously in nude mice after miR-1307-5p was stably inhibited. Tumor size on the 20th day; L (left) is lv-NC, R (right) is lv-miR-1307-5p-inhibitor

In the mouse tumorigenesis experiment, tumor dimensions were recorded every 5 days after inoculation and the tumor volume was calculated. After overexpression of miR-1307-5p, the tumorigenicity of A549 and H1299 was higher than that of the control group, with statistically significant difference (Fig. 6a, Additional file 4: Figure S4A); after inhibition of miR-1307-5p, the tumorigenicity of A549 and H1299 was lower than that of the control group, with statistically significant difference (Fig. 6b, Additional file 4: Figure S4B). Twenty days later, the mice were euthanized, and tumor weights were recorded. Tumor weight of the overexpressed miR-1307-5p group was significantly higher than that of the control group (Additional file 4: Figure S4C), while tumor weight of the miR-1307-5p group was significantly lower than that of the control group (Additional file 4: Figure S4D). Immunohistochemical staining showed that, compared with the negative control group, cytoplasmic TRAF3 tumor staining in the overexpression group was decreased and the number of stained cells was clearly significantly reduced; while in the inhibition group, cytoplasmic TRAF3 tumor staining was increased and the number of stained cells significantly increased (Additional file 5: Figure S5A, S5B). Consistent with previous in vitro studies, miR-1307-5p promotes proliferation of lung adenocarcinoma cells in vivo and binds to TRAF3.

## Discussion

Lung cancer, one of the malignant tumors with the highest morbidity and mortality worldwide, is highly invasive and metastatic. Late lung adenocarcinoma is treated mainly by surgery [37]. However, early or surgically treatable locally advanced lung adenocarcinoma still has a high recurrence rate and distant metastasis rate after surgery, while non-resectable and recurrent lung adenocarcinoma can only be treated with chemotherapy, with a poor prognosis and a low survival rate [29, 38–40]. Therefore, it is very important to study the molecular mechanism of malignant progression of lung adenocarcinoma to improve its treatment and prognosis.

Clinical and epidemiological studies have shown a strong link between chronic inflammation and cancer. Up to 20 percent of cancers are linked to chronic inflammation, and one-third of those are caused by smoking and inhaling pollutants [41, 42]. Cancer-related inflammation affects many aspects of malignant tumors, including proliferation and survival of malignant cells, angiogenesis, tumor metastasis, and tumor response to chemotherapy drugs and hormones, and may provide targets for the prevention and treatment of lung cancer [43, 44]. NF-κB is an important intracellular nuclear transcription factor, whose family includes NF-κB1 (p50), NF-κB2 (p52), RelA (p65), RelB, and c-rel [45, 46]. NF-κB plays an important role in inflammation and is an indispensable part of the process of tumorigenesis, which is closely related to inflammatory immunity, cell apoptosis, and tumor development [47]. Chronic NF-κB-driven airway inflammation promotes the production and maintenance of TGFβ/IL-10/retinoic acid-dependent FoxP3 + Tregs by selective activation of alveolar macrophages [48, 49]. However, the specific mechanism of inflammation regulating lung adenocarcinoma needs further study.

MiRNAs regulate a variety of biological signal pathways [50]. Abnormal expression of miRNAs is closely related to lung cancer [51]. More and more studies show that miRNAs play an important role in non-small cell lung cancer [52]. Therefore, our research group carried out some preliminary studies on miRNA in lung adenocarcinoma. The relationship between inflammation and cancer is a hot topic, and the research on the role of inflammation in the occurrence and development of lung adenocarcinoma is relatively less, while the role of miRNA in the regulation of inflammation and lung adenocarcinoma is even rare. The results of our preliminary lung adenocarcinoma function experiment showed that p65 could be used as an important candidate cancer gene. Through the second generation of high-throughput sequencing of Illumina, we screened out the miRNAs differential expression profile. In this study, miR-1307-5p with significant difference was selected from miRNAs differential expression spectrum. It was shown in vivo and in vitro that miR-1307-5p may be an important new target for the treatment of lung adenocarcinoma. Compared with normal lung cells and paracancerous tissues, miR-1307-5p is upregulated and promotes the proliferation of lung adenocarcinoma as shown by qPCR. Meanwhile, lung adenocarcinoma tissue microarray analysis (180 cores, 90 lung adenocarcinoma tissues, 90 paracancerous tissues; follow-up time: 2012–2017) of the correlation between miR-1307-5p and clinicopathological features showed that the increased expression of miR-1307-5p was significantly related to the decreased overall survival rate of patients with lung adenocarcinoma. In a series of functional experiments such as CCK-8, EdU, plate cloning, Transwell assay, and scratch assay, overexpression of miR-1307-5p significantly promoted the proliferation and migration of lung adenocarcinoma cells, inhibited the apoptosis, and migration of lung adenocarcinoma cells by miR-1307-5p. Furthermore, tumor volumes and weights in the lentiviral-miR-1307-5p group were significantly larger than in the lentiviral-NC group, while those in the lentiviral-miR-1307-5p-inhibitor group were significantly smaller than in the lentiviral-NC group. This series of functional experiments fully verified that miR-1307-5p promoted the proliferation and migration of lung adenocarcinoma.

In order to further study the mechanism of miR-1307-5p, the potential target of miR-1307-5p in lung adenocarcinoma was further studied. Bioinformatics analysis predicted that TRAF3 is a target gene of miR-1307-5p. The double luciferase test directly verified that miR-1307-5p targets TRAF3. NC, mimic, and inhibitor had no effect on the activity of the target gene mutation vector and empty vector, mimic had obvious inhibition on the activity of the target gene wild-type vector, but after inhibitor treatment, the activity of the target gene wild-type vector was improved to some extent; but after mutation site, mimic had no inhibition on the target gene vector, while after inhibitor treatment, the activity of mutant vector was improved. Therefore, miR-1307-5p targeted regulation of TRAF3.

The protein encoded by TRAF3 belongs to the TNF receptor-related factor (TRAF) protein family. TRAF3 is characterized by ubiquitin E3 ligase, which is mainly composed of the characteristic TRAF domain at the carboxyl end and the typical C3HC4 RING finger domain at the N end [53]. TRAF protein is related to the TNF receptor (TNFR) superfamily and mediates its signal transduction. This protein is involved in the signal transduction of CD40, a member of the TNFR family that is very important for the activation of the immune response [54]. The main downstream signal transduction events mediated by TRAF include the activation of NF-κB and MAPK [55]. In addition, some members of the TRAF family, especially TRAF2 and TRAF3, act as regulators of specific signal transduction pathways. TRAF3 can inhibit the activity of NF-κB by continuously mediating the degradation of NF-κB-induced kinase (Nik). The downregulation of TRAF3 causes the activation of the NF-κB pathway, while the upregulation of TRAF3 inhibits the activation of NF-κB pathway [56]. TRAF3 regulates the NF-κB pathway in several cancers. For example, overexpression of miR-17-92 can directly target TRAF3 to upregulate the NF-κB signaling pathway in gastric cancer; RIP2 has been identified as a new binding partner of TRAF3 in glioma and is involved in the regulation of NF-κB, and knocking down RIP2 can increase TRAF3 expression; and deletion of TRAF3 and CYLD in head and neck squamous cell carcinoma can activate NF-κB [57–60].

The NF-κB signaling pathway can be divided into classical and non-classical pathways. The most abundant form of NF-κB activated by pathological stimulation through a typical pathway is the p50:p65 heterodimer [61]. p65-mediated transactivation plays a key role in the pathogenesis of various types of chronic inflammation. Many inhibitors of upstream signaling molecules play a role in chronic inflammation due to the reduction of NF-κB p65 signaling [62]. A recent study has shown that both Tax-1 and tax-2 proteins encoded by the two genomes of HTLV form complexes with TNF receptor-related factor 3 TRAF3 and they found that the NF-κB pathway was activated in TRAF3-deficient cell lines; these results confirm that TRAF3 is required for the activation of NF-κB mediated by Tax, which is consistent with some research ideas and results in our study [63]. These studies show that the NF-κB pathway is activated by knocking down TRAF3. In this study, miR-1307-5p induced significant maladjustment of TRAF3 and affected the classical NF-κB pathway. MiR-1307-5p targeted to TRAF3 could degrade TRAF3 protein and activate phosphorylated p65, whereas inhibiting miR-1307-5p increased the protein levels of TRAF3 and inhibited phosphorylated p65.

TRAF3 plays a key role in the regulation of TLR4 signal transduction, and via the TLR4-MyD88-dependent pathway, downregulating TRAF3 can promote the activation of MAPK signal transduction by TLR4 and the phosphorylation of JNK and p38 [64, 65]. The MAPK pathway intersects with cell proliferation, stress, inflammation, differentiation, function synchronization, transformation, apoptosis, and other signal transduction pathways. ERK widely exists in various tissues and participates in the regulation of cell proliferation and differentiation; p38 mediates inflammation, apoptosis, and so on [66, 67]. It has been reported that capsaicin can induce the apoptosis of KSHV-positive primary lymphoma by inhibiting ERK and p38 MAPK signal transduction and IL-6 expression, inhibit ERK and p38 MAPK phosphorylation, and thus significantly inhibit the growth of primary lymphoma cells [68]. MMP-12 promotes the proliferation of macrophages and the secretion of IL-1βand IL-6 through ERK/p38 MAPK signaling pathway and induces the development of inflammation [69]. In this study, miR-1307-5p targeted TRAF3 to regulate the MAPK pathway, miR-1307-5p targeted to TRAF3 degraded TRAF3 protein and activated p-ERK1/2 and p-p38 proteins in the MAPK pathway to promote the proliferation of lung adenocarcinoma, while there was no significant change in ERK1/2 and p38 protein; however, inhibiting miR-1307-5p increased the protein levels of TRAF3 and inhibited the proteins p-ERK1/2 and p-p38 in the MAPK pathway to induce the apoptosis of lung adenocarcinoma, and there was no significant change in ERK1/2 and p38 protein. It can be seen that miR-1307-5p plays an indispensable role in promoting the proliferation of lung adenocarcinoma.

As shown through a series of in vitro and in vivo experiments, miR-1307-5p promotes the proliferation of lung adenocarcinoma, and miR-1307-5p targeting TRAF3 upregulates the MAPK/NF-κB pathway. MiR-1307-5p may thus be involved in the molecular mechanism of lung cancer development and may be a potential diagnostic and prognostic biomarker in lung adenocarcinoma.

## Conclusions

In conclusion, miR-1307-5p plays an important role in promoting the development of lung adenocarcinoma, including cell growth, proliferation, and invasion. We demonstrated that miR-1307-5p targets TRAF3 and activates NF-κB/MAPK pathway to promote the proliferation of lung adenocarcinoma. Therefore, the miR-1307-5p/TRAF3/NF-κB/MAPK axis may be a new target for the treatment of human lung adenocarcinoma and may be further identified as a potential prognostic factor in the future.

## Supplementary information

**Additional file 1: ** Fig S1.

**Additional file 2:** Fig S2.

**Additional file 3:** Fig S3.

**Additional file 4:** Fig S4.

**Additional file 5:** Fig S5.

## Data Availability

All the data and materials were available under the agreement of the authors.
