# Supplementary material for: MiR-1307-5p targeting TRAF3 upregulates the MAPK/NF-κB pathway and promotes lung adenocarcinoma proliferation
Source: Cancer Cell Int. 2020 Oct 12;20:502. doi: 10.1186/s12935-020-01595-z (PMC7552495; doi:10.1186/s12935-020-01595-z)
Supplement: Supplementary file 1 — Additional file 1: Fig S1. [file 12935_2020_1595_MOESM1_ESM.docx]

Figure S1


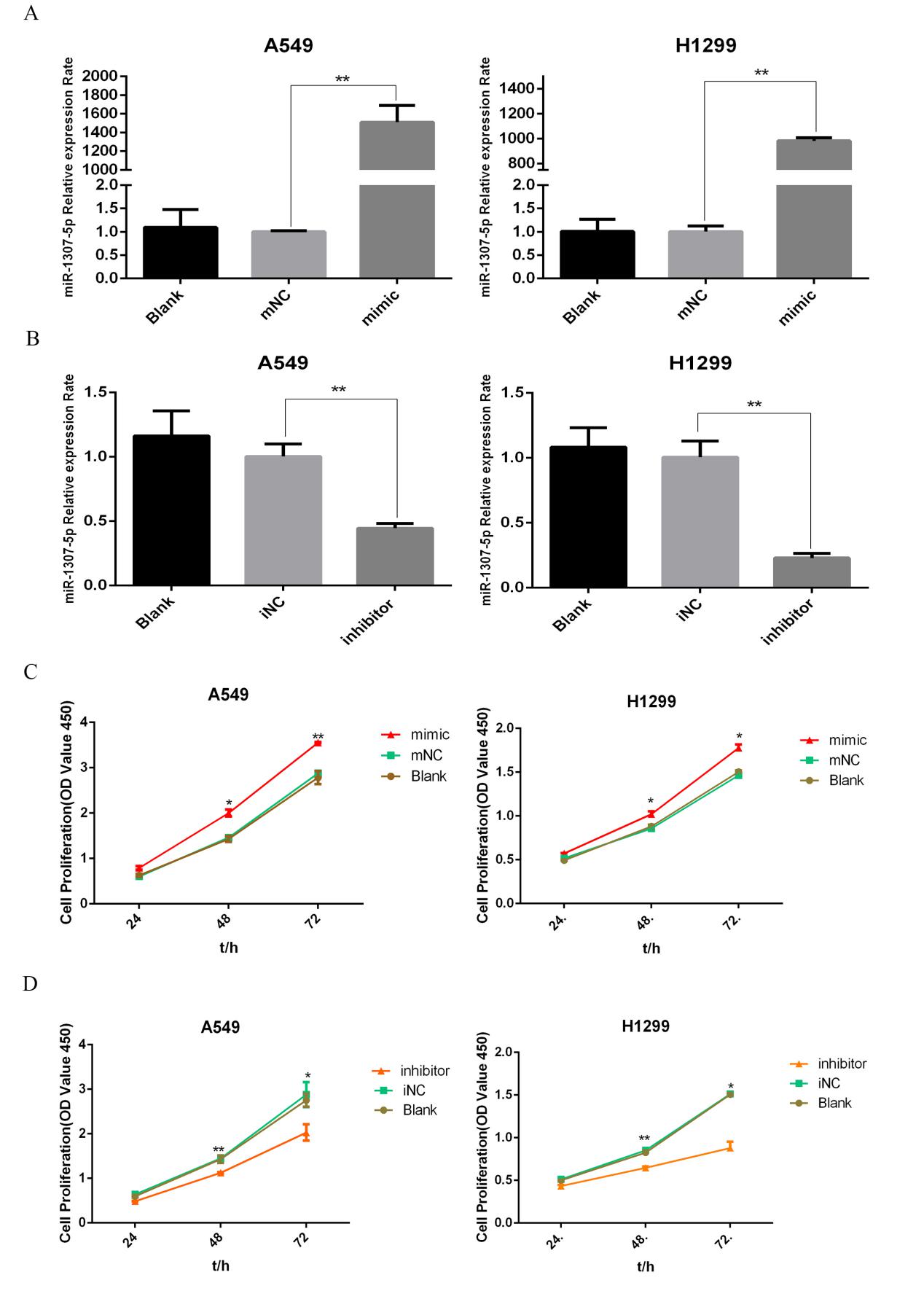


Figure S1. **MiR-1307-5p promotes the proliferation of LUAD cells.** MiR-1307-5p promotes the proliferation of LUAD cells. (A, B) QPCR was used to detect the transient efficiency of miR-1307-5p between treatment groups. Compared with the control group, * P < 0.05 and ** P < 0.01. (C, D) The CCK-8 method was used to detect the proliferation of H1299 and A549 lung adenocarcinoma cells after transient transfection. Overexpression of miR-1307-5p promoted cell viability (C), while knockdown of miR-1307-5p inhibited cell viability (D). Compared with the control group, * P < 0.05 and ** P < 0.01. Data are expressed as mean ± standard deviation. The experiment was repeated three times.
