# Supplementary material for: MiR-1307-5p targeting TRAF3 upregulates the MAPK/NF-κB pathway and promotes lung adenocarcinoma proliferation
Source: Cancer Cell Int. 2020 Oct 12;20:502. doi: 10.1186/s12935-020-01595-z (PMC7552495; doi:10.1186/s12935-020-01595-z)
Supplement: Supplementary file 2 — Additional file 2: Fig S2. [file 12935_2020_1595_MOESM2_ESM.docx]

Figure S2


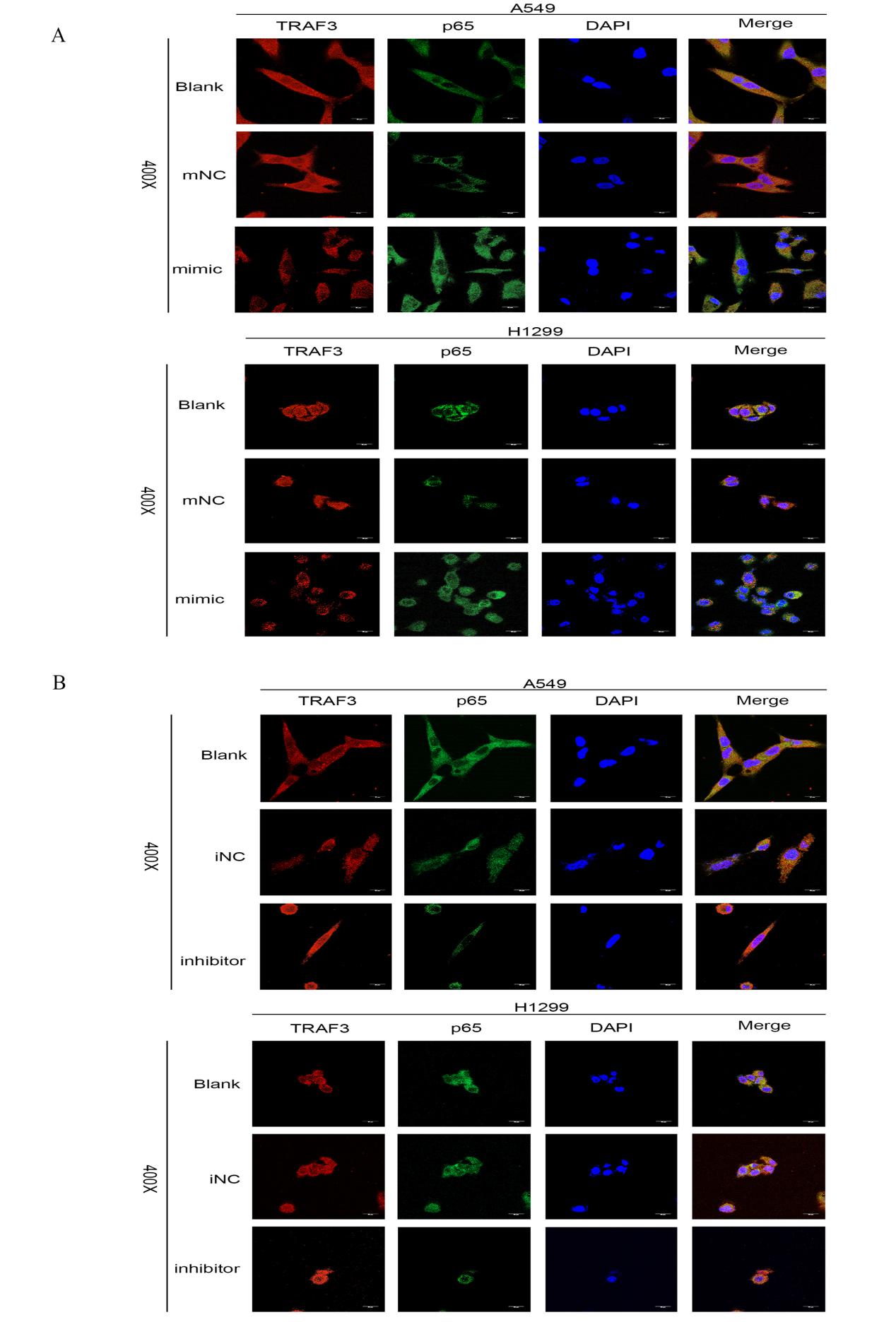


Figure S2. **Effect of miR-1307-5p-targeted TRAF3 on NF-κB pathway.** (A, B) Immunofluorescence assay was used to detect TRAF3 (red), p65 (green), and DAPI (blue) in each treatment group after transient transfection of H1299 and A549 lung adenocarcinoma cells. The co-localization of p65 and TRAF3 was significant. Compared with the control group and the blank group, lung adenocarcinoma cells in the mimic group showed lower expression of cytoplasmic TRAF3 while p65 fluorescence was enhanced and expressed in both the cytoplasm and the nucleus (A). Conversely, TRAF3 fluorescence was relatively increased in the inhibitor group and expressed in the cytoplasm and partially in the nuclei, while p65 fluorescence was decreased and only partially expressed in the cytoplasm (B). Data are expressed as mean ± standard deviation. The experiment was repeated three times.
