# Supplementary material for: MiR-1307-5p targeting TRAF3 upregulates the MAPK/NF-κB pathway and promotes lung adenocarcinoma proliferation
Source: Cancer Cell Int. 2020 Oct 12;20:502. doi: 10.1186/s12935-020-01595-z (PMC7552495; doi:10.1186/s12935-020-01595-z)
Supplement: Supplementary file 3 — Additional file 3: Fig S3. [file 12935_2020_1595_MOESM3_ESM.docx]

Figure S3


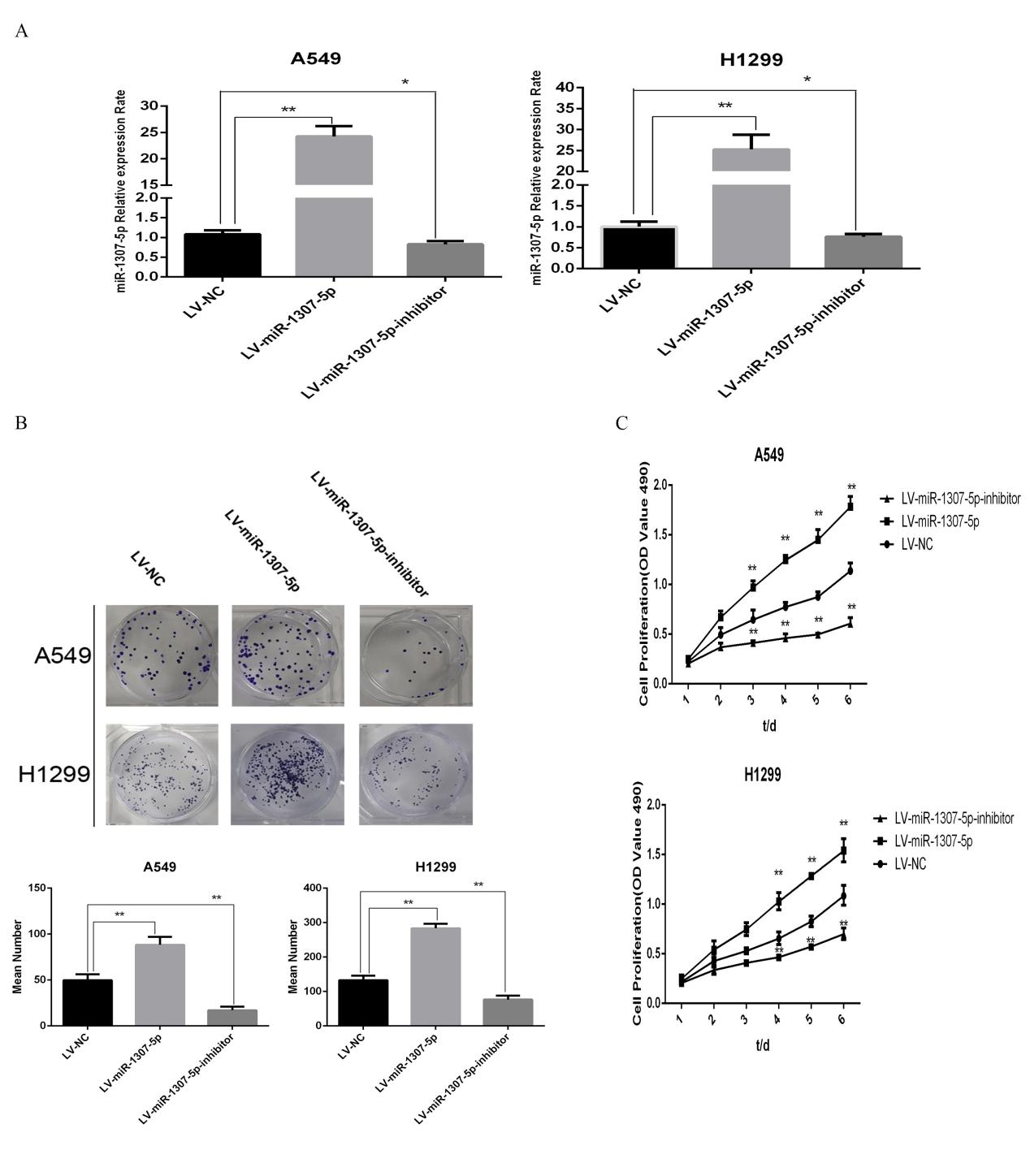


Figure S3. **MiR-1307-5p promoted the proliferation of stable lentivirus strains of LUAD.** (A) The stable rotation efficiency of miR-1307-5p in each treatment group. Compared with the control group, * P < 0.05 and ** P < 0.01. (B) Plate cloning test was used to detect the proliferation of each treatment group. Overexpression of miR-1307-5p promoted cell proliferation, while knockdown of miR-1307-5p inhibited cell proliferation. Compared with the control group, * P < 0.05 and ** P < 0.01. (C) CCK-8 method was used to detect the proliferation of miR-1307-5p in H1299 and A549 lung adenocarcinoma cells. Overexpression of miR-1307-5p promoted cell proliferation, while knockdown of miR-1307-5p inhibited cell proliferation. Compared with the control group, * P < 0.05 and ** P < 0.01. Data are expressed as mean ± standard deviation. The experiment was repeated three times.
