# Supplementary material for: MiR-1307-5p targeting TRAF3 upregulates the MAPK/NF-κB pathway and promotes lung adenocarcinoma proliferation
Source: Cancer Cell Int. 2020 Oct 12;20:502. doi: 10.1186/s12935-020-01595-z (PMC7552495; doi:10.1186/s12935-020-01595-z)
Supplement: Supplementary file 4 — Additional file 4: Fig S4. [file 12935_2020_1595_MOESM4_ESM.docx]

Figure S4


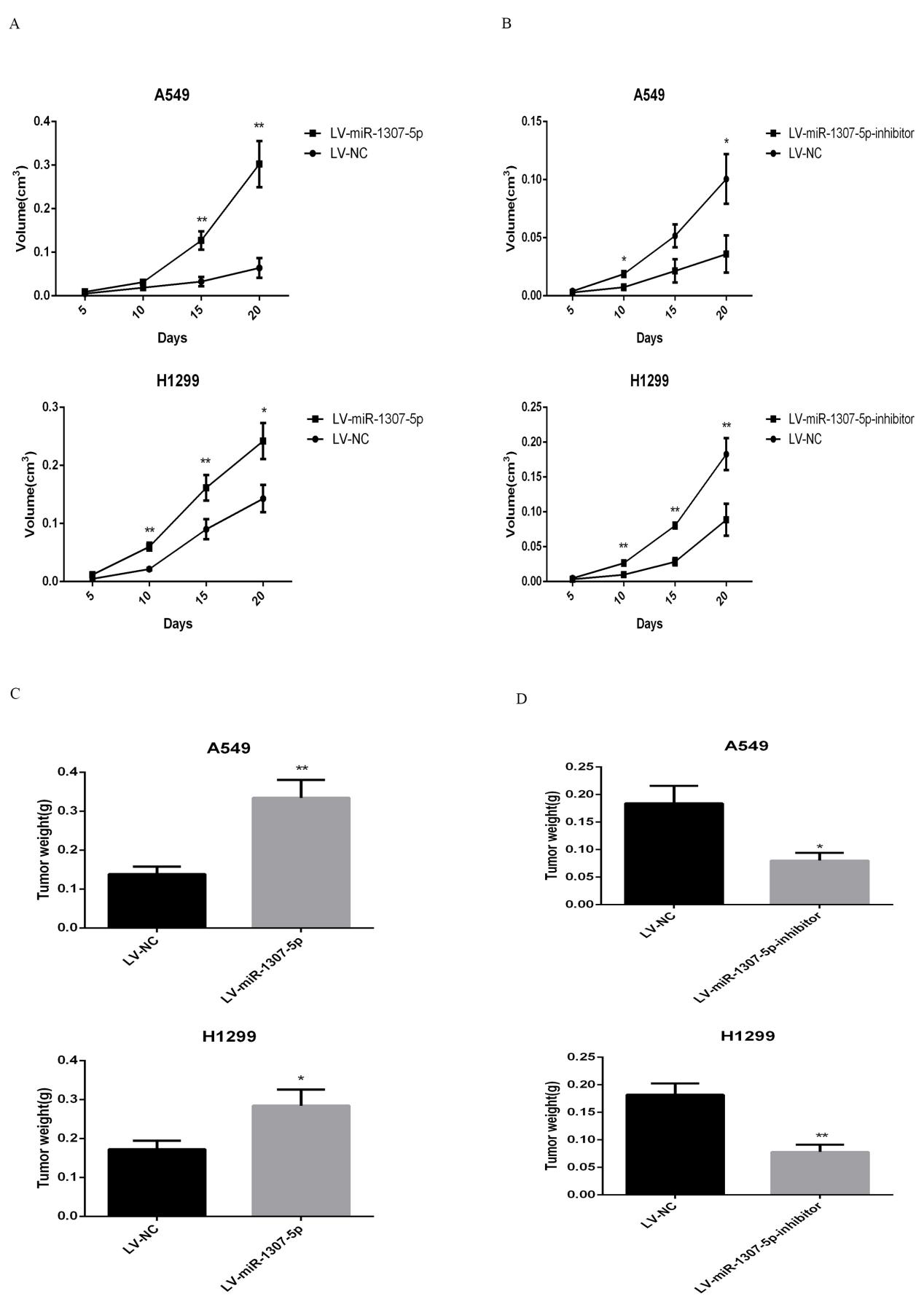


Figure S4. **MiR-1307-5p promotes tumor growth *in vivo*.** (A) The tumor growth curve of stable overexpression of miR-1307-5p within 20 days. After overexpression of miR-1307-5p, A549 and H1299 had significantly increased tumorigenicity in vivo. Compared with the control group, * P < 0.05 and ** P < 0.01. (B) The tumor growth curve of miR-1307-5p was inhibited stably within 20 days. After inhibition of miR-1307-5p, A549 and H1299 were significantly weakened in vivo. Compared with the control group, * P < 0.05 and ** P < 0.01. (C) Tumor weight in nude mice. The tumor weight of the miR-1307-5p group was significantly higher than that of the control group. Compared with the control group, * P < 0.05 and ** P < 0.01. (D) The weight of subcutaneous tumor in nude mice. The tumor weight of the miR-1307-5p group was significantly lower than that of the control group. Compared with the control group, * P < 0.05 and ** P < 0.01. Data are expressed as mean ± standard deviation. The experiment was repeated three times.
