# Supplementary material for: MiR-1307-5p targeting TRAF3 upregulates the MAPK/NF-κB pathway and promotes lung adenocarcinoma proliferation
Source: Cancer Cell Int. 2020 Oct 12;20:502. doi: 10.1186/s12935-020-01595-z (PMC7552495; doi:10.1186/s12935-020-01595-z)
Supplement: Supplementary file 5 — Additional file 5: Fig S5. [file 12935_2020_1595_MOESM5_ESM.docx]

Figure S5


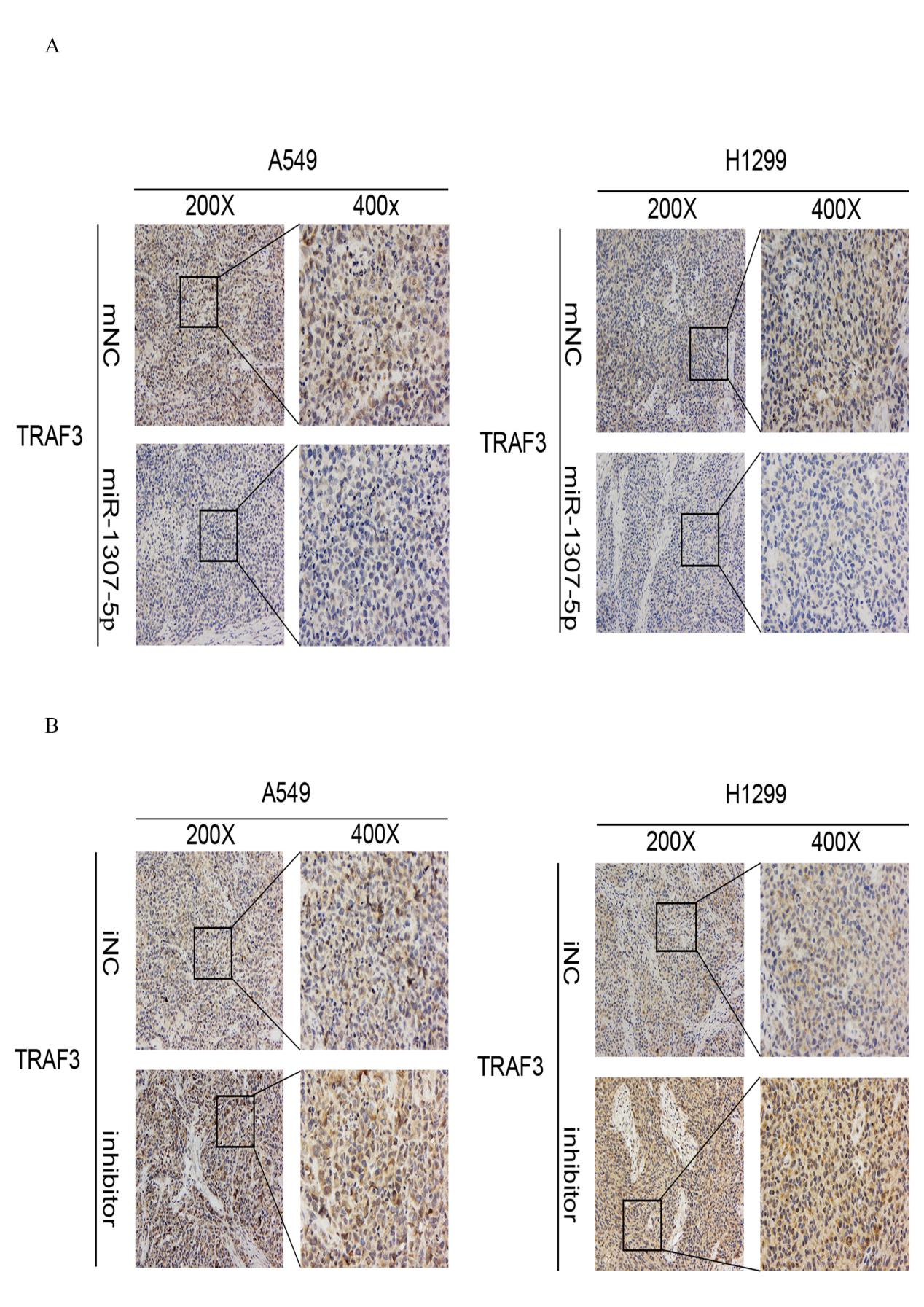


Figure S5. **The expression of TRAF3 protein was detected by immunohistochemistry.** (A,B) The expression of TRAF3 protein was detected by immunohistochemistry. Compared with the negative control group, staining of TRAF3 protein in the cytoplasm of the tumor in the overexpression group was weakened, and the number of stained cells was significantly reduced (A); staining of the tumor in the inhibition group was enhanced, and the number of stained cells was significantly increased (B). Data are expressed as mean ± standard deviation. The experiment was repeated three times.
